# PD16 - Prevalence of childhood food allergy in Canada: a focus on under-represented populations

**DOI:** 10.1186/2045-7022-4-S1-P16

**Published:** 2014-02-28

**Authors:** Lianne Soller, Moshe Ben-Shoshan, Megan Knoll, Daniel Harrington, Joseph Fragapane, Lawrence Joseph, Yvan St Pierre, Sebastien La Vieille, Kathi Wilson, Susan Elliott, Ann Clarke

**Affiliations:** 1 McGill University, Montreal, Canada; 2 University of Toronto, Toronto, Canada; 3 Health Canada, Ottawa, Canada; 4 University of Waterloo, Waterloo, Canada

## Background

Studies suggest individuals of low socioeconomic status (SES), immigrants, and Aboriginal peoples, may have fewer food allergies than the general population. However, given the difficulty in recruiting such populations using conventional survey methodologies, the prevalence of food allergy among these populations in Canada has not been estimated.

## Objectives

To compare the prevalence of food allergies among children from low-income, immigrant and Aboriginal populations to children from the general Canadian population.

## Methods

Using 2006 Canadian Census data, postal codes with high proportions of low-income, immigrant, and Aboriginal populations were extracted and households randomly selected to participate in a telephone survey. Information on food allergies and demographic data was collected for all children (defined as below 18 years of age). Food allergy was defined according to self-report. Prevalence estimates were weighted using Census data to account for our targeted sampling.

## Results

Between September 2010 and September 2011, 12,747 households were contacted to complete the survey, of which 6,403 responded (50.2% response rate), representing 3,271 children. Among all children, the prevalence of allergy to any food was 7.49% (95% Confidence Interval (CI), 5.93, 9.05). Children born in Canada had considerably more food allergies than those born elsewhere [7.96% (95% CI, 6.24, 9.68) versus 3.26% (95% CI, 1.46, 5.07)]. The prevalence was higher for children residing in households above the low income cut-off (LICO) than below the LICO [7.81% (95% CI, 5.48, 10.14) versus 6.24% (95%CI, 4.12, 8.36)], and for children with versus without Aboriginal ancestry [7.62% (95% CI, 5.98, 9.26) versus 6.03% (95% CI, 1.30, 10.76)]; however, these differences were not statistically significant due to overlapping confidence intervals.

## Conclusions

Our study found that immigrant children experience fewer food allergies than Canadian-born children. Although the data suggest a trend towards a lower prevalence of food allergy among low-income and Aboriginal children, wide confidence intervals preclude definitive conclusions.

